# IgG4-related ophthalmic disease presenting as posterior scleritis in a pediatric patient

**DOI:** 10.1186/s12348-025-00459-9

**Published:** 2025-03-27

**Authors:** Irmak Karaca, Albert John Bromeo, Azadeh Mobasserian, Amir Akhavanrezayat, Charles DeBoer, Zheng Xian Thng, Jia-Horung Hung, Woong-Sun Yoo, Anadi Khatri, Negin Yavari, Ba Trung Nguyen, Dalia El Feky, Cigdem Yasar, Osama Elaraby, Aim-On Saengsirinavin, Xiaoyan Zhang, Frances Andrea Anover, Ankur Sudhir Gupta, Diana V. Do, Christopher Or, Quan Dong Nguyen

**Affiliations:** 1 Spencer Center for Vision Research, Byers Eye Institute, School of Medicine, Stanford University, 2370 Watson Court, Palo Alto, CA USA; 2 Moran Eye Center, University of Utah, Salt Lake City, UT USA; 3 Asian Eye Institute, Makati, Philippines; 4 National Healthcare Group Eye Institute, Tan Tock Seng Hospital, Singapore, Singapore; 5 Birat Aankha Aspatal, Biratnagar, Nepal

**Keywords:** Posterior scleritis, IgG4-related ophthalmic disease, Infliximab, Pediatric

## Abstract

**Purpose:**

To report IgG4-related ophthalmic disease (IgG4-ROD) presenting as posterior scleritis in a pediatric patient.

**Observations:**

A 7-year-old girl presented with proptosis, painful eyelid swelling, and restricted extraocular movements (EOM) of her left eye (OS). Visual acuity (VA) was 20/20 in right eye (OD) and counting fingers (CF) at 1 foot in OS. Slit lamp examination revealed 2 + anterior chamber (AC) cells, optic disc edema (ODE) with elevated appearance of macula in OS. Optical coherence tomography (OCT) showed significant subretinal fluid (SRF) in macula, B-scan ultrasound (US) demonstrated T-sign in OS. Orbital MRI was also consistent with posterior scleritis and periorbital inflammation. Extensive systemic work-up was unremarkable. Thus, the patient was started on intravenous methylprednisolone (IVMP) 30 mg/kg/day for 3 days, along with topical therapy in OS, which led to an improvement of proptosis, EOM restriction, AC cells, as well as ODE and SRF in macula in OS. Fluorescein angiography (FA) showed leakage from optic disc in OS. The patient was then switched to oral prednisone with slow tapering and started on methotrexate (MTX). Given the recurrence of proptosis and painful eyelid swelling on systemic steroid tapering, serum IgG4 levels were ordered and found to be elevated at 149.9 mg/dL (range, 1–99). Therefore, the patient was diagnosed as ‘possible’ IgG4-ROD *(based on diagnostic criteria)* and started on infliximab (7.5 mg/kg) and IVMP monthly infusions with continuation of MTX 20 mg weekly and slower tapering of oral prednisone, which led to resolution of clinical findings, improvement of VA to 20/20 in OS.

**Conclusion and importance:**

Posterior scleritis may be the initial presentation of IgG4-ROD in children. Refractory course is not uncommon. Biologics are effective in the long-term control of inflammation.

## Introduction

Posterior scleritis is characterized by inflammation of the sclera posterior to the insertion of the rectus muscles and accounts for 2–12% of all scleritis cases in adults [1–4]. It is mostly seen in female patients in their 4th decade, and composes a clinical challenge given its rare presentation and sight-threatening complications. In contrast, pediatric scleritis (1.2% of all scleritis) more frequently presents with posterior scleral involvement as compared to adults [5]. Cheung and Chee.

described the largest series of posterior scleritis in the pediatric population (20 eyes of 13 patients) with the median age of 12 years [6]. Common features included concurrent anterior uveitis, optic disc edema, and retinal striae. B-scan ultrasound demonstrating T-sign was the most useful confirmatory investigation. Visual prognosis is usually good, with 92.6% of eyes recovered with a visual acuity (VA) of ≥20/40 and a final median VA of 20/20. In addition, systemic association is rare in pediatric posterior scleritis [7]. 

IgG4-related disease (IgG4-RD) is an increasingly recognized chronic, multisystem inflammatory disorder with numerous presentations [8, 9]. IgG4-RD is now more recognized as responsible for a significant proportion of orbital inflammatory disease which was previously labeled as idiopathic orbital inflammation or reactive lymphoid hyperplasia [10]. IgG4-related ophthalmic disease (IgG4-ROD) is a specific term for the orbital and adnexal involvement in IgG4-RD which is one of the most common disease presentation. The lacrimal gland is the most frequently involved ocular structure in IgG4-ROD, though lymphoplasmacytic infiltration of IGG4 plasma cells can be seen in almost any tissue of the orbit and adnexa including the trigeminal nerve, extraocular muscles, orbital fat, and eyelids [11, 12]. 

Herein, we aimed to report a pediatric patient with ‘possible’ IgG4-ROD presenting as posterior scleritis and required therapy with infliximab and methotrexate. To the best of our knowledge, the index case is the first case of IgG4-ROD presenting as posterior scleritis in a pediatric patient.

## Case report

**Fig. 1:**
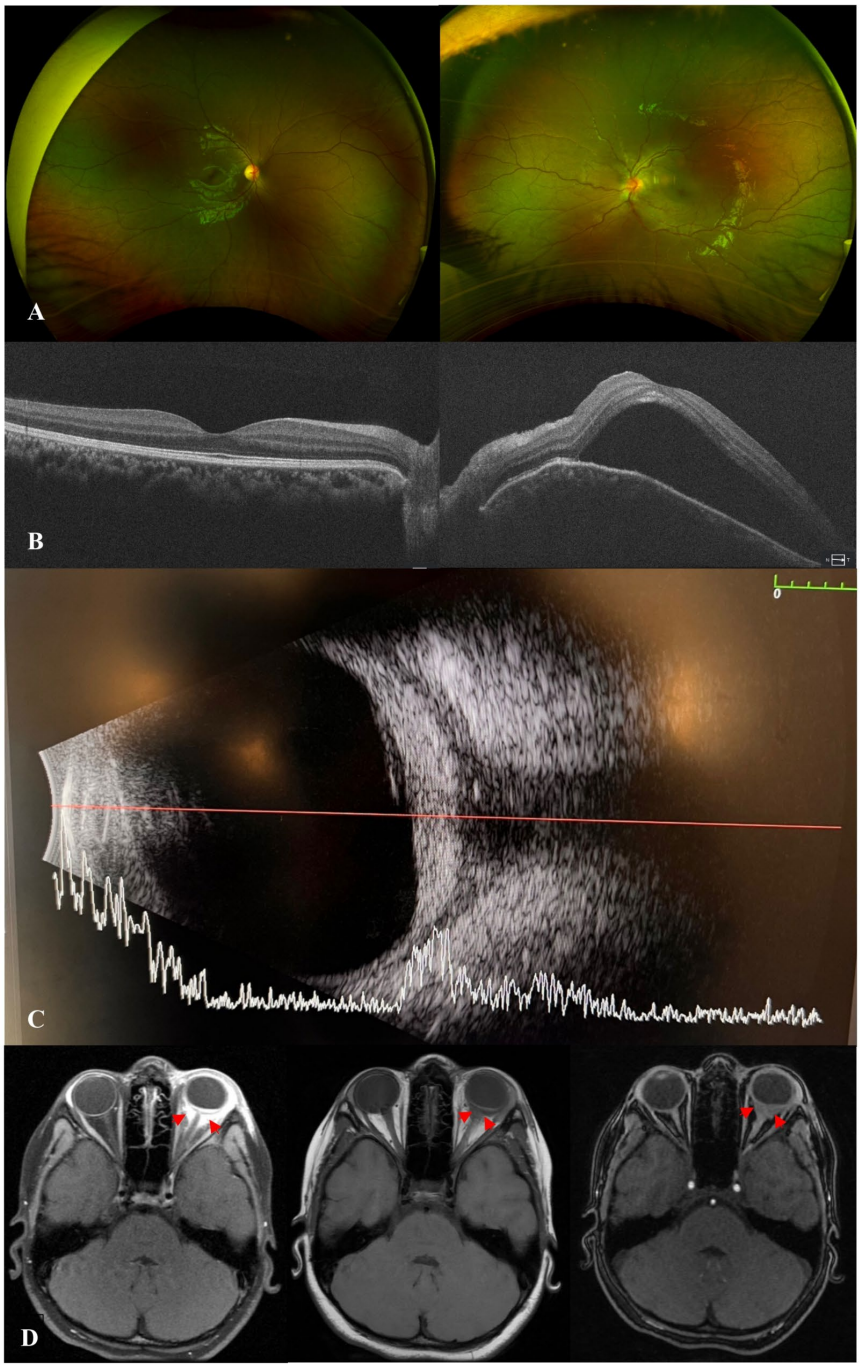
Initial presentation of the patient with optic disc edema in left eye (**A**) and subretinal fluid at macula (**B**), as well as T-sign on B-scan (**C**) and MRI showing T2 and T1 hypointense and hyper-enhancing tissue along the left posterior retina and along the posterior aspect of the globe extending into the intraconal fat (**D**, red arrows)

**Fig. 2:**
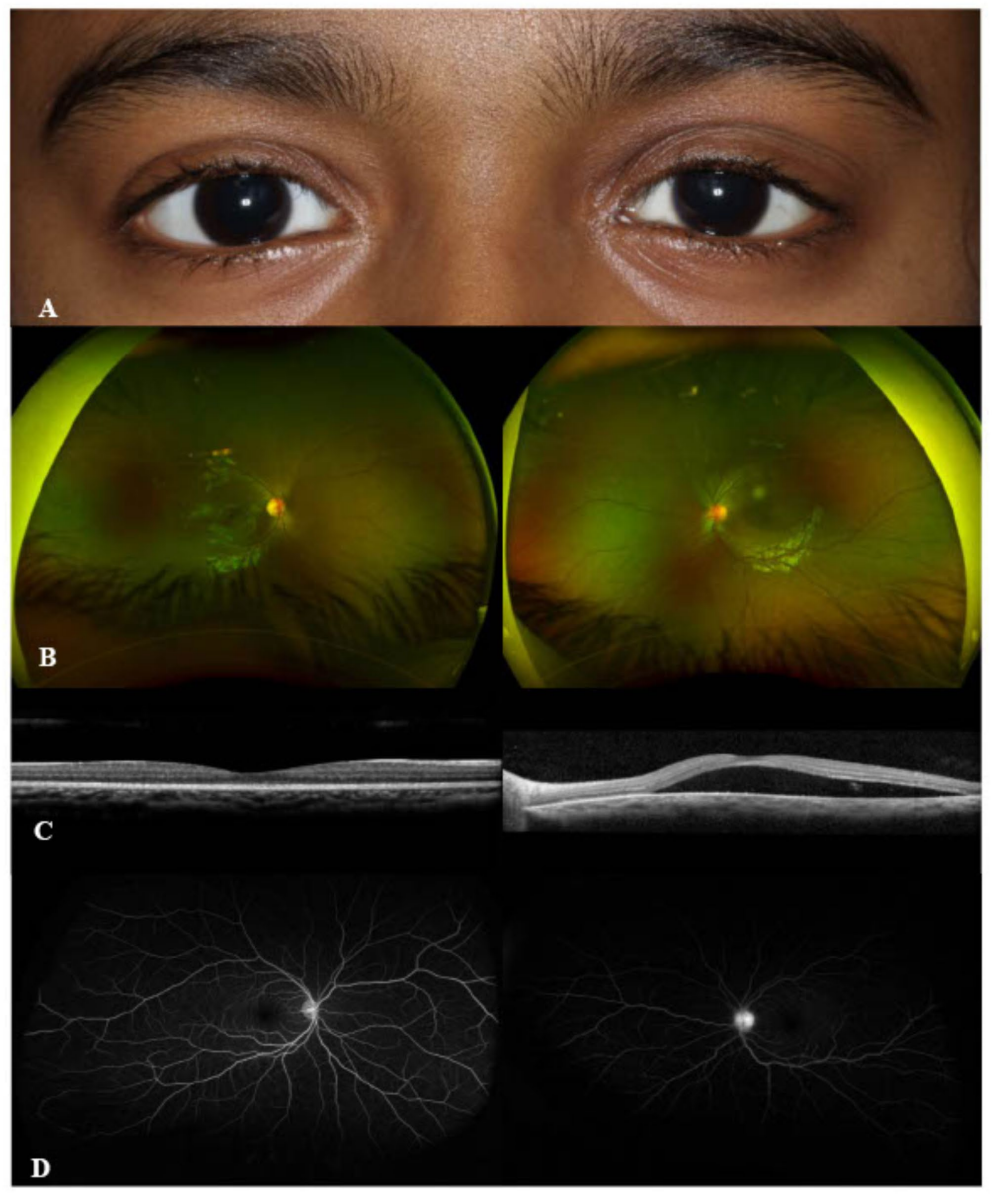
Following pulse intravenous methylprednisolone therapy, improved proptosis (**A**), optic disc edema (**B**), subretinal fluid at macula on optical coherence tomography (**C**) in the left eye along with leakage from optic disc on fluorescein angiography (**D**)

**Fig. 3:**
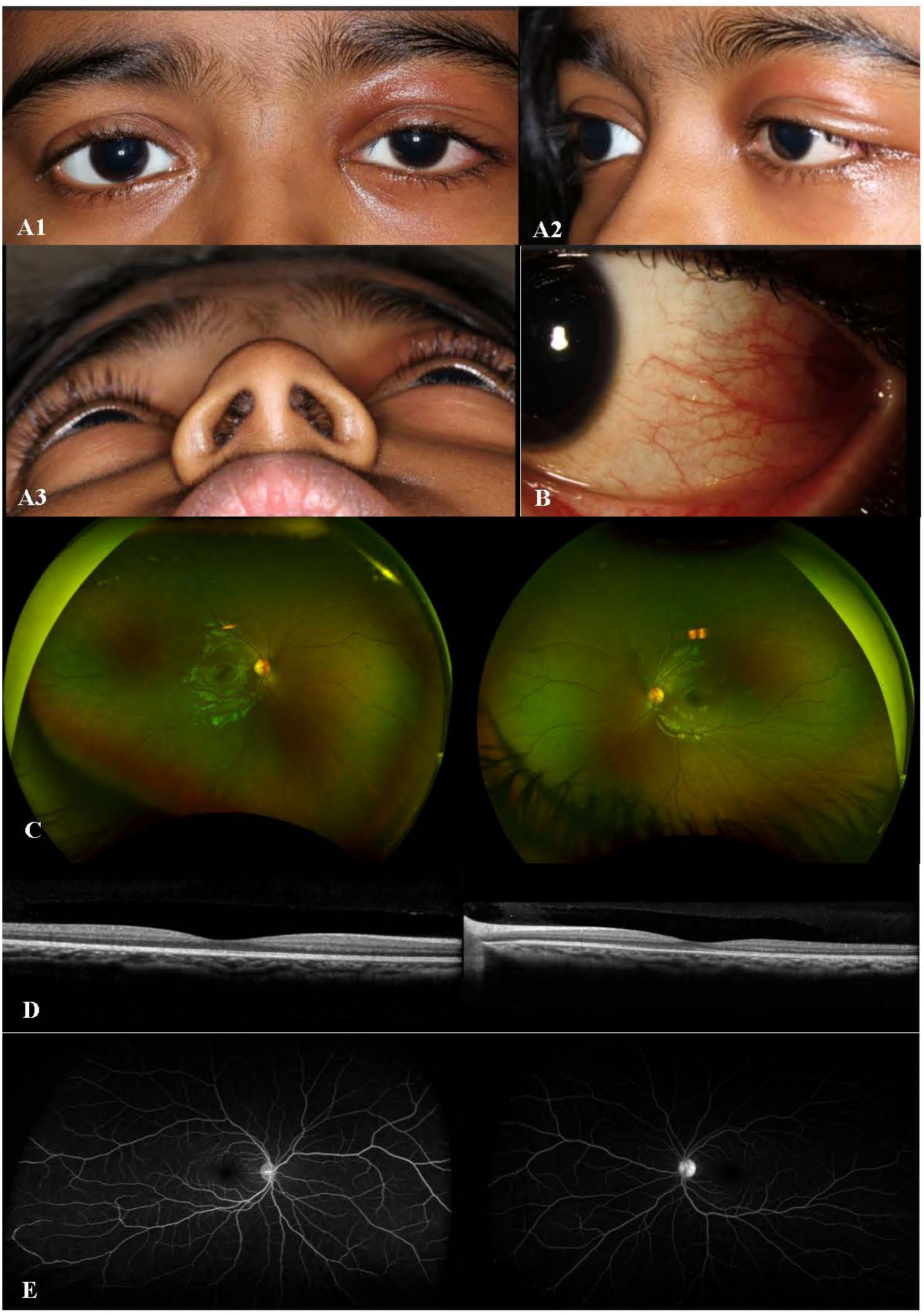
At 3 months of follow-up, while on methotrexate therapy and oral prednisone tapering, recurrence of proptosis, periorbital edema and erythema (**A**), anterior scleral inflammation (**B**), despite unremarkable fundus exam (**C**) and optical coherence tomography (**D**) with stable mild leakage from optic disc in the left eye on fluorescein angiography (**E**)

**Fig. 4:**
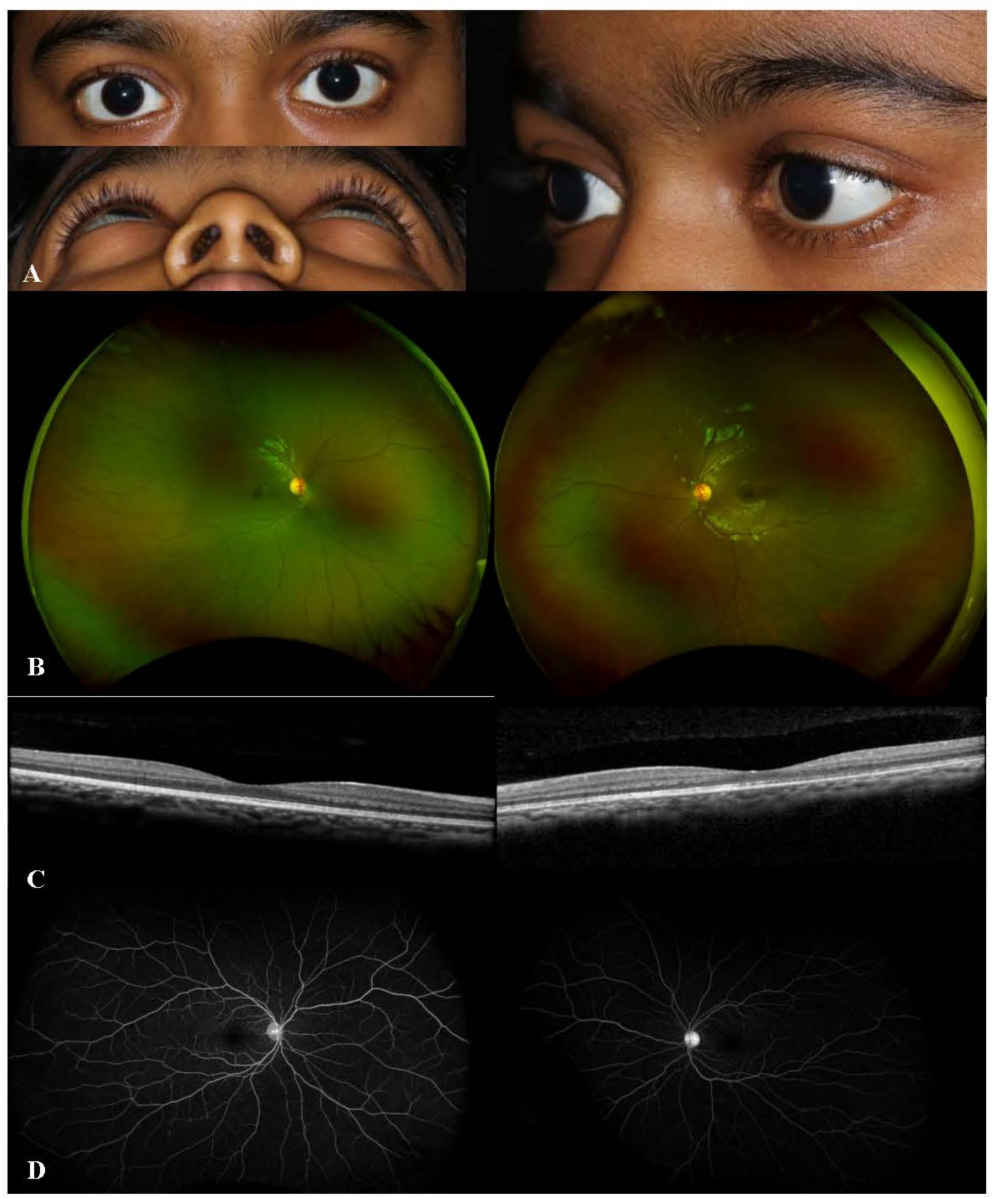
After first cycle of infliximab and intravenous methylprednisolone therapy, improved proptosis (**A**) with unremarkable fundus exam (**C**) and optical coherence tomography (**D**) as well as improved leakage from optic disc in the left eye on fluorescein angiography (**E**)

**Fig. 5:**
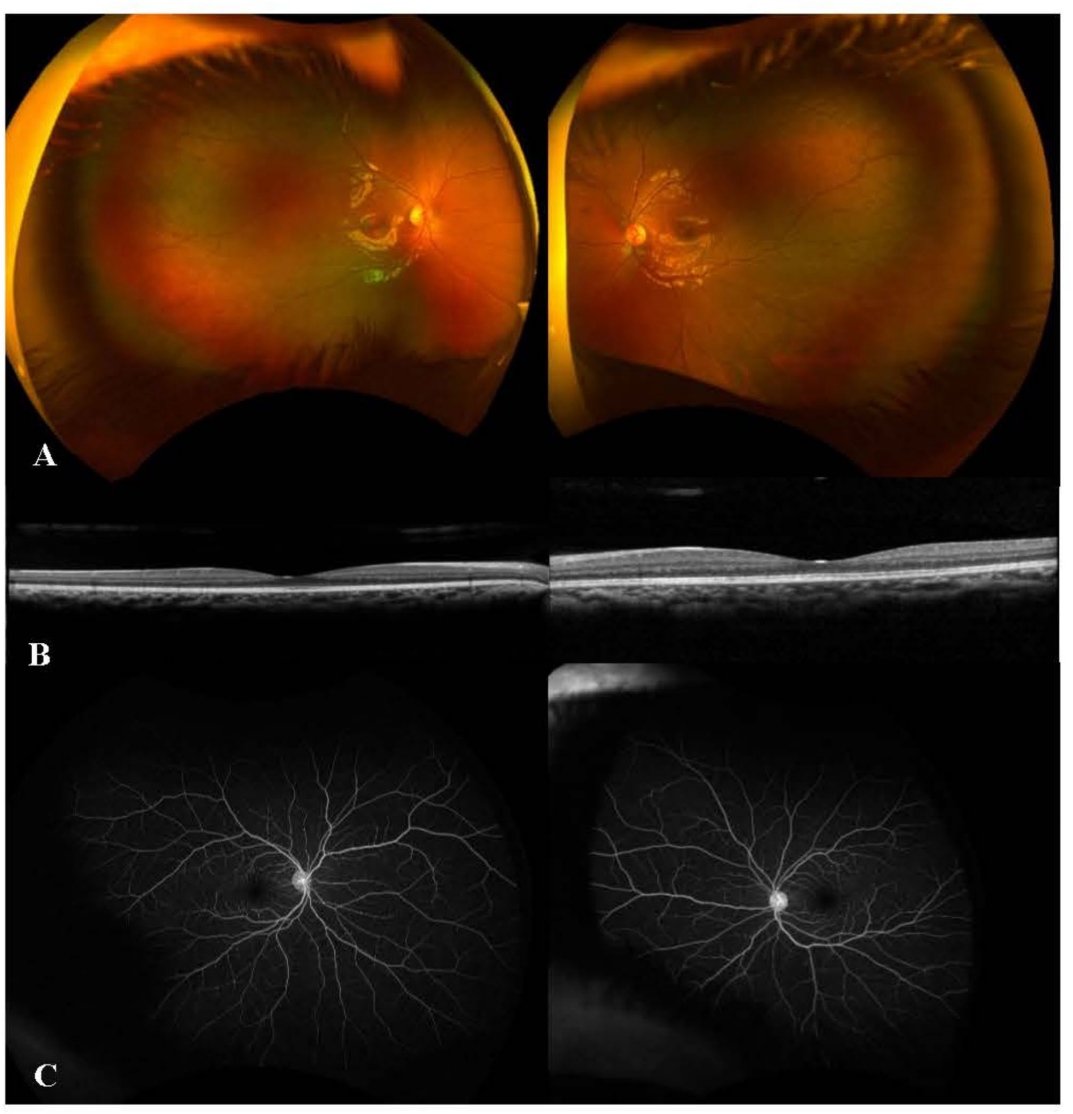
After five cycles of infliximab and intravenous methylprednisolone therapy, unremarkable fundus exam (**A**), optical coherence tomography (**B**) and fluorescein angiography (**C**) in both eyes

A 7-year-old girl presented with painful eyelid swelling and decreased vision in the left eye (OS). The medical history was unremarkable except for a history of blunt trauma at a swing set when the swing hit the left side of her face about 2 weeks prior to presentation. VA was 20/20 in right eye (OD) and counting fingers (CF) at 1’ in OS. IOP was 9 mmHg in OD and 13 mmHg in OS. There was significant proptosis as well as mild restriction of extraocular movements (EOMs) in OS. Slit-lamp examination (SLE) was unremarkable in OD, while the presence of 2 + anterior chamber (AC) cells was noted in OS. Fundus examination revealed optic disc edema (ODE) with elevated appearance of macula in OS (Fig. 1A). Optical coherence tomography (OCT) showed significant subretinal fluid (SRF) in macula (Fig. 1B) as well as increased peripapillary retinal nerve fiber layer (RNFL) thickness consistent with ODE in OS. B-scan ultrasound (US) demonstrated T-sign in OS (Fig. 1C). MRI demonstrated T2 and T1 hypointense and hyper-enhancing tissue along the left posterior retina and along the posterior aspect of the globe extending into the intraconal fat, concerning for posterior scleritis and periorbital inflammation in the intraconal fat and along the orbital segment of the optic nerve (Fig. 1D). In addition, asymmetric enhancement of the left lacrimal gland was noted. The patient was afebrile with no evidence of systemic infection and had no joint symptoms or rashes. Additional work-up showed elevated erythrocyte sedimentation rate (ESR) (32 mm/h) and C-reactive protein (CRP) (1.7 mg/L) levels, and remaining testing were unremarkable including complete blood count, complete metabolic panel, urinalysis, syphilis, HIV, QuantiFERON, HSV/VZV/CMV PCR, Toxoplasma, Lyme, Bartonella, chest X-ray, ACE, lysozyme, ANA, anti-dsDNA, ANCA, proteinase-3 and myeloperoxidase antibodies, C3 and C4. The patient was diagnosed with posterior scleritis in OS and subsequently started on intravenous methylprednisolone 30 mg/kg/day for 3 days, along with prednisolone acetate 8 times daily and cyclopentolate 2 times daily in OS, which led to improvement of proptosis, EOM restriction (Fig. 2A), as well as AC cells, ODE and SRF in macula in OS (Fig. 2B and C). Fluorescein angiography (FA) performed following resolution of periorbital swelling showed optic disc leakage in OS (Fig. 2D). The patient was then switched to oral prednisone 30 mg daily (1 mg/kg/day) with slow tapering and started on methotrexate (MTX) 15 mg weekly along with folic acid supplementation considering the severe course of ocular inflammation. At 1.5 months of follow-up, VA improved to 20/30 in OS with resolution of EOM restriction and proptosis. AC was quiet and there was a complete resolution of SRF in OS. However, FA showed persistence of leakage from the optic disc along with mild peripheral retinal vascular leakage in OS. Thus, MTX was increased to 20 mg weekly; oral prednisone 10 mg daily and prednisolone acetate twice daily in OS were continued with further tapering and cyclopentolate was discontinued in OS. At 2.5 months of follow-up, the patient once again presented with painful eyelid swelling in OS, when she was at MTX 20 mg weekly and oral prednisone 10 mg daily, and later improved with the increment of oral prednisone to 30 mg daily and stable dose of methotrexate 20 mg weekly. However, three weeks later, her symptoms worsened again with severe painful eyelid swelling in OS, when she was on MTX 20 mg weekly, oral prednisone 20 mg daily, and prednisolone acetate 2 times daily. VA was 20/20 in both eyes (OU). The patient had recurrence of proptosis (Hertel_*(108 mm)*_ = 18 mm in OD, 21 mm in OS) (Fig. 3A_1 − 3_), as well as mildly restricted EOMs in OS. SLE revealed mild scleral injection temporally (Fig. 3B) and 0.5 + AC cells in OS. Fundus examination was unremarkable, OCT did not show SRF, and FA was stable as mild leakage from the optic disc in OS (Fig. 3C-E). Systemic evaluation did not show any evidence of infection. However, CRP level was elevated further to 2.8 mg/L. Given the concern for chronic orbital inflammation in OS, serum immunoglobulin G (IgG) (total + subgroups) levels were ordered, which revealed elevated serum IgG4 levels at 149.9 mg/dL (range, 1–99). The patient was diagnosed as ‘possible’ IgG4-related ophthalmic disease (IgG4-ROD) [13]. Immunomodulatory therapy (IMT) was escalated and she was started on infliximab (IFX) 7.5 mg/kg/month along with IV methylprednisolone (IVMP) 30 mg/kg/day for 3 days a month, and continued MTX 20 mg weekly with slower tapering of oral prednisone 20 mg daily and prednisolone acetate 2 times daily. After first cycle of IFX and IVMP therapy (Fig. 4), CRP returned to normal range (< 0.3 mg/L). IgG4 level was also reduced even though slightly elevated at 122.1 mg/dL. Follow-up MRI after 2 cycles of IFX and IVMP therapy showed complete resolution of left uveoscleral enhancement and left ocular proptosis, as well as marked decrease in left retrobulbar and left optic nerve sheath enhancement. Whole body PET/CT scan was also performed to investigate other tissue site involvement which revealed no evidence of metabolically active inflammatory processes; specifically, no evidence of FDG avid activity involving the left orbit or the vasculature. At the latest follow-up (after 5 cycles of IFX therapy), VA was 20/20 in OU without proptosis or EOM restriction. SLE and fundus examination were unremarkable in OU including OCT (Fig. 5). FA showed resolution of optic disc leakage in OS. The patient was planned to continue IFX and IVMP infusions, as well as MTX 20 mg weekly and continued tapering of oral prednisone 7.5 mg daily.

## Discussion

In this report, we described the first reported case of IgG4-ROD presenting as posterior scleritis along with chronic orbital inflammation in a pediatric patient which required aggressive immunomodulatory therapy.

IgG4-RD was first implicated and recognized by Hamano et al. [8] in 2001 in a group of patients with “*autoimmune pancreatitis with elevated serum IgG4 levels”*. In 2012, nomenclature was standardized as “IgG4-RD” which involved tumefactive tissue lesions, usually but not always, accompanied by increased IgG4 serum levels [10]. Histopathology shows lymphoplasmacytic infiltrates with predominance of IgG4-positive plasma cells, fibrosis (usually in a storiform pattern), obliterative phlebitis, and eosinophils. Both humoral and cellular immunity have been thought to play a role in disease pathogenesis [11, 12]. Thus far, several diseases, some with orbital manifestations, were recognized as part of spectrum of IgG4-RD including Mikulicz disease [14]. Despite the rare incidence of IgG4-RD (0.28–1.08/100,000 in Japanese population), IgG4-ROD incidence accounts for 4–34% of total IgG4-RD cases. Moreover, it is not uncommon to find no abnormalities in other organs at the time the ocular lesion is diagnosed. The lacrimal gland is the most involved site (62–88%) in IgG4-RD, followed by the pancreas [15, 16]. Male-to-female ratio is reported as 1.3:1 with the mean age of onset as 55.5±12.9 years. Common patterns of involvement in IgG4-ROD are described as ‘dacryoadenitis’, ‘enlarged orbital nerves’, ‘orbital fat involvement’ and ‘sclerosing orbital inflammation without lacrimal gland involvement’. In 2015, diagnostic criteria for IgG4-ROD were defined by the Japanese Study Group for IgG4-Related Ophthalmic Disease as: (1) Imaging studies show enlargement of the lacrimal gland, trigeminal nerve, or extraocular muscle as well as masses, enlargement, or hypertrophic lesions in various ophthalmic tissues, (2) Histopathologic examination shows marked lymphocyte and plasmacyte infiltration, and sometimes fibrosis. A germinal center is frequently observed. IgG4 + plasmacytes are found and satisfy the following criteria: ratio of IgG4 + cells to IgG + Cells of 40% or above, or more than 50 IgG4 + cells per high-power field (x400), (3) Blood test shows elevated serum IgG4 (≥135 mg/dl) [13]. Diagnosis is classified as ‘‘definitive’’ when (1), (2), and (3) are satisfied; ‘‘probable’’ when (1) and (2) are satisfied; and ‘‘possible’’ when (1) and (3) are satisfied. Recently, the authors reported that the most severe symptom of ophthalmic lesions is visual loss due to optic neuropathy [17]. The diagnostic criteria was revised in 2023 accordingly by drawing attention to the potential presence of optic neuropathy in IgG-ROD which causes visual acuity and field deterioration; as well as careful differentiation of lymphomas, other than mucosa-associated lymphoid tissue (MALT) lymphoma, as they may also contain IgG4 + cells [18]. 

IgG4-ROD is a rare and relatively new entity, which can be challenging to diagnose, particularly in case with atypical features such as scleritis. There are few case reports which have introduced IgG4-RD as an emerging cause of idiopathic scleritis [19–25]. Additionally, scleritis was reported in 4 (1%) patients with IgG4-ROD in the recent series by Goto et al. [17] Inflammatory tissue biopsies and serum IgG4 levels are the only warrant of a timely and accurate diagnosis of IgG-ROD. Although serum IgG4 level is the most common laboratory test performed to diagnose IgG4-RD, a raised serum IgG4 is *not* present in up to 30% of patients, or may also be high in other diseases [12, 26]. Thus, it has been thought that serum IgG4 levels is more useful for monitoring response to treatment, if found elevated at initial diagnosis. In our patient, IgG4-ROD was highly suspected based on the concurrent chronic orbital inflammation which was accompanied by high serum IgG4 levels and enhancement of the left lacrimal gland on MRI. No definitive/probable diagnosis could be made by the biopsy of the sclera or extraocular tissues because of procedural difficulty also considering very young age of the patient. Thus, our patient was diagnosed as ‘possible’ IgG4-ROD.

IgG4-RD may also involve other tissues, and progression to a chronic state is not uncommon. Whole body PET/CT scan has been indicated and recommended as the most useful modality for detecting multi-organ involvement and staging [12]. In addition, early and aggressive treatment is crucial given the likelihood of irreversible organ damage when left untreated. IgG4-RD is characterized by excellent response to systemic corticosteroids as a mainstay therapy; however, repeated relapses usually occur (67%) following tapering. In our patient, improvement of symptoms following systemic corticosteroid therapy with prompt recurrence with tapering were also supportive of the diagnosis. IMT such as azathioprine, MTX, mycophenolate mofetil, cyclophosphamide, adalimumab, and IFX have been used in the management of IgG4-ROD for steroid-sparing effect [27]. Considering IgG4-producing plasma cells in the histopathology, clinical response of IgG4-RD has also been highly successful with rituximab therapy [12, 28, 29]. Low dose radiotherapy was also used in small number of cases with IgG4-ROD [30]. Similarly, we started the patient on MTX and then escalated to IFX therapy. Additionally, IMT is also often required in the management of pediatric posterior scleritis (~ 50%) [6], and IFX has also been successfully used for this purpose [31–33]. 

Interestingly, review of literature demonstrates some differences of IgG4-RD in children compared to adult population [34–37]. Ocular involvement is the most common presentation of IgG4-RD in children, and the frequency of ophthalmic manifestations are higher in children. Moreover, the pattern of ocular involvement differs in which orbital soft tissue and extraocular muscles are the most common affected sites, whereas the lacrimal glands are very infrequently described to be involved. IgG4-ROD is more commonly seen in girls, and usually presents as unilateral disease. Main presenting symptoms include orbital swelling, eyelid tenderness and proptosis. All these features of pediatric IgG4-ROD were also observed and recognized in our patient. Children usually have the localized form (1 organ involvement) of IgG4-RD, and extra-ophthalmic manifestations are rarely seen. Consistent with the literature, there was no other organ involvement in our patient on whole body PET/MRI. In addition, serum IgG4 levels may be normal (30–70%), and normal levels of acute phase reactants is not uncommon (≈ 30%). Currently, there is no diagnostic criteria available, nor treatment guidelines or randomized treatment trials on how to manage IgG4-RD in children.

In conclusion, we reported a unique case of atypical presentation of IgG4-ROD with posterior scleritis as initial disease and concurrent chronic orbital inflammation. Despite a very rare incidence, IgG4-RD should be kept in our armamentarium in the differential diagnosis of scleritis including posterior scleritis in pediatric population, and serum IgG4 level is warranted as a part of the assessment of scleritis. Considering normal serum IgG4 levels in 30% of patients, histopathological analysis should be performed when appropriate to confirm the diagnosis of IgG4-RD. Prompt and accurate diagnosis of IgG4-RD is highly important to initiate the proper therapy, to search for other organ involvement, and to improve the clinical course given the chronicity of the disease which will also be helpful in preventing devastating visual morbidity.

## Data Availability

No datasets were generated or analysed during the current study.
